# Lenalidomide in the Treatment of Chronic Lymphocytic Leukemia

**DOI:** 10.1155/2012/393864

**Published:** 2012-05-08

**Authors:** Agostino Cortelezzi, Mariarita Sciumè, Gianluigi Reda

**Affiliations:** Hematology and Transplantation Unit, Foundation IRCCS Ca' Granda Ospedale Maggiore Policlinico and Università degli Studi Di Milano, 20122 Milan, Italy

## Abstract

The application of nucleoside analogue-based chemotherapy and immunotherapy with rituximab or alemtuzumab has increased both response rate and survival in patients with Chronic Lymphocytic Leukemia (CLL). However, because none of these therapies is curative, sequential therapeutic regimens are required. The majority of patients with relapsed or refractory CLL carry poor prognostic factors and show shorter overall survival and resistance to standard treatment. Numerous drugs have recently been approved for CLL therapy and many novel agents are under clinical investigation. The role of the tumor microenvironment and of immune dysfunction in CLL have allowed to enlarge the therapeutic armamentarium for CLL patients. This article will provide a comprehensive summary regarding mechanism of action, efficacy and safety of lenalidomide in CLL patients. Relevant clinical trials using lenalidomide alone or in combinations are discussed. Lenalidomide shows good activity also in relapsed/refractory or treatment-naive CLL patients. Definitive data from ongoing studies are needed to validate overall and progression-free survival. The toxicity profile might limit lenalidomide use because it can result in serious side effects, but largely controlled by gradual dose escalation. Further understanding of the exact mechanism of action in CLL will allow more efficacious use of lenalidomide alone or in combination regimens.

## 1. Introduction

Chronic lymphocytic leukemia (CLL) shows a remarkable heterogeneity in its clinical course, from long-term survival to fast progression and early death. Previously treated patients are known to have poor overall prognosis. In such cases, the disease almost invariably becomes resistant to various subsequent chemotherapies, leading to more toxicities and deterioration of quality of life. 

Fludarabine given with cyclophosphamide and rituximab is considered the cornerstone of CLL treatment, but this effective chemoimmunotherapy cannot be given with the same success to all patients. The majority of patients with relapsed or refractory CLL carry poor prognostic features, like, deletion (17p) or TP53 mutation, which are strong predictors of shorter overall survival and resistance to first-line treatment, particularly fludarabine-based regimens [1, 2].

The management of chronic lymphocytic leukemia is currently undergoing profound changes through the introduction of new therapeutic and diagnostic tools. 

A common feature of CLL patients is impairment of the immune system with hypogammaglobulinemia, defective function of B, T, NK cells and defective antigen presentation [3]. Immunomodulatory drugs, such as, lenalidomide, represent a promising approach for relapsed/refractory or treatment-naïve patients, because they show antitumor activity and promote immunostimulation.

## 2. Mechanisms of Action of Lenalidomide in CLL

The precise anti-CLL mechanism of action of lenalidomide is not yet completely defined. Potential mechanisms of action include antiangiogenic effect, blockade of protumor cytokines, inhibition of prosurvival interaction between bone-marrow stromal cells and CLL cells, and enhancement of T helper and cytotoxic T cells function [4]. 

Immunomodulatory effects of lenalidomide include a costimulatory effect on T-cell responses by increasing IL-2 and INF-gamma secretion and subsequently proliferation of IL-2-activated T cells. Effects of lenalidomide in the production of cytokines is shown by increased circulating cytokines levels, particularly, IL-6, IL-10, IL-2, and TNF-receptor-1 levels; while it is a potent inhibitor of tumor necrosis factor alpha (TNF-alfa), this inhibition may results from increased degradation of TNF-alfa mRNA [5, 6]. Data from LeBlanc suggest that lenalidomide activates T lymphocytes directly by increased phosphorylation of CD28 and increased transcriptional activity of AP-1 and indirectly by enhancing immune synapses between antigen-presenting cells and effector T cells [7–9]. 

Lenalidomide exposure is able to lead to immune synapses between CLL cells and T cells and promotes costimulatory activation of B cells. During treatment with lenalidomide in CLL patients an increase in circulating Ig levels has been observed. This increased production of Igs may be explained by enhanced B-cell costimulatory activity via activation of lymphocytes through phosphoinositide-3-kinase dependent upregulation of CD154 (CD40L) on CLL cells, as described by Lapalombella et al. [10]. 

The immune activation of CLL cells with subsequent upregulation of costimulatory molecules, such as, CD40, CD80 and CD86, might be responsible for Tumor Flare observed in CLL patients treated with lenalidomide [6]. Lenalidomide has also been shown to activate NK T cells; one of its postulated mechanisms of action is increased antibody-dependent cellular cytotoxicity (ADCC). IL-2-activated T cells are able to activate NK cells enhancing tumor cell death [5, 7, 11]. 

Many tumors, including CLL, are characterized by increased number of T regulatory cells and expression of CD152 (CTLA4) in T cells with a correlation with advanced disease and adverse prognostic factors. Lenalidomide reduces T regulatory cell proliferation and suppression function [12]. 

Lenalidomide also shows antiangiogenic properties in vitro. However, Andritsos et al. [13] reported that VEGF serum concentrations remained unchanged in patients treated with lenalidomide. Furthermore, Ferrajoli et al. [6] did not observe changes in neovascularization in the bone marrow biopsies in patients treated with lenalidomide. 

## 3. Clinical Results Utilizing Lenalidomide in CLL

### 3.1. Clinical Studies with Lenalidomide as Single Agent in CLL

The second-generation immunomodulatory agent lenalidomide has shown considerable activity in CLL, either alone or in combinations and both as first-line and salvage treatment. 

Activity in CLL was first demonstrated by Chanan-Khan et al. [14] in a nonrandomized phase II study that included patients with relapsed or refractory B CLL. In this trial 25 mg of lenalidomide was administered every 21 days of a 28-day cycle in 45 CLL patients. Patients were to continue treatment until disease progression, unacceptable toxicity, or complete remission. The major overall response rate (ORR) in this study was 47%, with a complete response (CR) rate of 9%. Tumor lysis syndrome (TLS) and tumor flare reaction (TFR) occurred in 5% and 58% patients, respectively. TLS is characterized by electrolyte imbalance, uremia, and renal failure, while TFR is associated with painful swelling of lymph nodes and/or splenomegaly with sometimes fever and skin rash. 

Because of toxicity, including tumor lysis syndrome in two of the first 29 patients, the dosing schedule was modified and the new treatment schema stipulated a dose escalation beginning at 5 mg/day and a target dose of 25 mg/day. None of the patients (with or without the prophylaxis) required interruption, discontinuation, or dose reduction of therapy because of flare reaction. 

Together with TFR, fatigue (83%) was the most common nonhematologic adverse event reported. Grades 3 or 4 neutropenia, thrombocytopenia, and anemia occurred in 70%, 45%, 18% patients during therapy, respectively. There were six episodes of febrile neutropenia. Although neutropenia was observed in a subset of patients, neither opportunistic nor bacterial infections were problematic in this heavily pretreated group of patients. Pulmonary embolism occurred in two patients.

In another clinical trial of lenalidomide in pretreated CLL patients [6], 44 patients received lenalidomide continuously at 10 mg/day with a dose escalation up to 25 mg/day. Treatment was discontinued if disease progression or excessive toxicity was observed. In this pretreated group of patients, ORR was 32%, with 7% achieving a CR. The most common toxicity was myelosuppression, and the median daily dose of lenalidomide tolerated was 10 mg. Severe neutropenia, thrombocytopenia, and anemia were reported in 41%, 15%, and 3% of patients. None of the 44 patients had grade 3 or 4 episodes of tumor lysis; however, the incidence of TFR at any grade was higher in patients with lymph nodes larger than 5 cm (53%); grade 3 tumor flare reaction was reported in 2% of the courses and grade 1 or 2 tumor flare was reported in 10% of the courses. The most common infection was pneumonia, complicating 3% of the courses. FUO was observed in 2% of the courses. The median daily dose of lenalidomide tolerated by the patients in this study was 10 mg, and only 3 patients (7%) were able to tolerate the dose of 25 mg daily for at least 1 month. Myelosuppression was the most frequent reason for treatment interruption and dose reduction. Nonhematologic toxicity consisted of grade 1 to 2 fatigue, observed in 22% of the courses and grade 3 to 4 in only 1%. Only one case of deep vein thrombosis was reported, suggesting that this complication may be more common when lenalidomide is used in association with steroids and in patients with plasma cell dyscrasias. 

Treatment with lenalidomide was associated with an ORR rate of 31% in patients with 11q or 17p deletion, of 24% in patients with unmutated VH, and of 25% in patients with fludarabine-refractory disease. 

Sher et al. [15] reviewed the cases of relapsed/refractory CLL patients with high-risk cytogenetics who were included in the trial of Chanan-Khan et al. [14]; clinical response was reported in 38% of these patients, with 19% CR. 

The question of whether continuous exposure is superior to the 3 weeks on/1 week off schedule reported by Chanan-Khan et al. [14] will require further investigation.

While data from Ferrajoli et al. [6] and Chanan-Khan et al. [14] proposed an escalation dose up to 25 mg, a report from Andritsos et al. [13] described serious adverse events four patients with relapsed CLL treated with lenalidomide (25 mg/d for 21 days of a 28-day cycle). Tumor flare was observed in three patients and was characterized by dramatic and painful lymph node enlargement resulting in hospitalization of two patients, with one fatal outcome. Another patient developed sepsis and renal failure. 

Chen et al. [16] reported results of lenalidomide therapy in 25 previously untreated patients. Lenalidomide dosing involved an escalation starting from a dose of 10 mg up to 25 mg/day. Serious toxic complications (tumor lysis and fatal sepsis) occurred in the first two patients, the protocol was modified with a 2,5 mg starting dose and an escalation up to 10 mg as target dose. Twenty-two patients were treated with this schema. The ORR was 56%, no CR. TFR was evident in 88% of patients, mostly low grade. 

In a phase II study Aue et al. [17] reported a change in administration schedule of lenalidomide, which was given in pulse dosages for 3 weeks followed by 3 weeks off. This study involved high-risk pretreated patients with a poor prognosis: 52% was Rai stage III-IV, 43% had del(17p), and 64% expressed unmutated IgVH genes. Lenalidomide was administered at 10 mg daily for 21 days of a 42-day cycle, for a total of 4 cycles. Grade 3-4 neutropenia, thrombocytopenia, and anemia occurred in 56%, 30%, and 15% of cycles, respectively. No TLS cases were seen. However, the hypothesis of achieving a safer and more tolerable toxicity profile was not obtained, since TFR was observed in 78%, 48%, 38%, and 30% in 1st, 2nd, 3rd, and 4th cycle, respectively. ORR was only 16% but patients with del(17p) and bulky disease appeared to have a remarkable PR rate of 80%

Badoux et al. [18] investigated lenalidomide for elderly untreated CLL patients. Lenalidomide was administered to sixty patients 65 years of age and older at 5 mg daily with a possible dose escalation of 5 mg every 28 days up to 25 mg daily. At a median followup of 29 months, 53 patients (88%) were alive and 32 patients (53%) remained on therapy. Estimated 2-year progression-free survival was 60%. The overall response rate to lenalidomide therapy was 65%, including 10% CR, 5% CR with residual cytopenia. Neutropenia was the most common grade 3 or 4 treatment-related toxicity observed in 34% of treatment cycles. Major infections or neutropenic fever occurred in 13% of patients. There were no grade 3 or 4 episodes of tumor flare or any tumor lysis syndrome in this study. Also compared with baseline levels, the authors noted an increase in serum immunoglobulin levels across all classes.

According to these several studies, TFR is a common toxicity described with lenalidomide. Because of its high incidence, its considerable morbidity and its clinical presentation resembling disease progression, an early diagnosis, and an accurate management are critical for effective use of lenalidomide in patients with CLL. 

Often TFR included a sudden onset of painful and tender enlargement of disease-involved lymph nodes, the spleen, and/or liver, which was frequently accompanied by low-grade fever, localized erythema, or generalized rash (often diffuse, erythematous, nonpruritic, and maculopapular) and occasionally associated with bone pain. Patients who develop a TFR tend to have a higher stage of disease, but mostly there is no significant difference noted with regard to the incidence of TFR among patients with bulky versus nonbulky disease. 

The severity of TFR can be graded according to the National Cancer Institute Common Toxicity Criteria [i.e. grade 1, mild pain not interfering with function grade 2, moderate pain (pain or analgesics interfering with function, but not interfering with activities of daily living); grade 3, severe pain (pain or analgesics interfering with function and activities of daily living); grade 4, disabling pain]. 

The identification and careful characterization of a TFR are important in the treatment of CLL patients with lenalidomide to avoid unnecessary morbidity or the premature discontinuation of effective therapy. It's recommended close monitoring of patients with CLL for signs of TFR, especially during the first days of lenalidomide therapy and treatment with an NSAID,such as, ibuprofen (at a dose of 400–600 mg every 6 hours), with steroid use considered only in cases of more intense TFRs.

 A slow-dose escalation strategy may reduce the intensity of TFR and should be considered, such as, reported by Chanan-Khan et al. [19] Eventually, dose adjustment of lenalidomide can be use in case of severe TFR, the dose can be increased again once the TFR subsides.

Chanan-Khan et al. [19] observed that prophylaxis with prednisone decreased the severity but not the incidence of TFR. Low-dose oral prednisone (20 mg daily for 5 days followed by 10 mg for 5 days) was used as TFR prophylaxis from treatment on days 1 to 10 of cycle 1, TFR prophylaxis was not given in subsequent cycles. Although exact clinical impact of TFR remains uncertain, the authors noted that the intensity of the TFR appeared to be correlated with a higher probability of achieving a CR; despite a higher CR rate their analysis did not demonstrate any benefit in the PFS in the TFR group.  Table 1 summarizes the results from clinical trials using lenalidomide alone in CLL patients.

### 3.2. Lenalidomide in Combination in CLL

Lenalidomide has been shown to activate NK cells and one of its postulated mechanisms of action is increased antibody-dependent cellular cytotoxicity (ADCC). Thus, lenalidomide would seem an appealing therapeutic agent to add to rituximab treatment, which is known to induce ADCC of CD-20-expressing CLL cells [20]. However a recent laboratory study suggested a potential antagonism between these two agents if used simultaneously with CLL cells, since lenalidomide down-regulated CD20, with a reduction in NK mediated ADCC of rituximab-treated CLL cells [21]. 

Clinical trials report that combined treatment with lenalidomide and rituximab improves activity and decreases the toxicity of lenalidomide. Ferrajoli et al. [22] investigated the combination lenalidomide plus rituximab in 60 patients with relapsed or refractory CLL. They treated patients with rituximab weekly for 1 cycle and then once every 4 weeks during subsequent cycles. Lenalidomide was administered at dose of 10 mg daily starting on day 9 of cycle 1 and continuing daily for 12 cycles. The ORR was 64%, with 8% CR. Data suggest that this combination is superior to the single-agent lenalidomide. The most frequently observed toxicity was neutropenia, occurring in 68% of patients. Twenty-two patients experienced low-grade tumor flare.

Frontline lenalidomide plus rituximab in patients with CLL was reported by James et al. [23] in 37 patients. Lenalidomide was started at a dose of 2,5 mg daily with an escalation up to 5 mg and 10 mg on day 8, if tolerated, every 21 days of a 28-day cycle for a total of 7 cycles. Patients received lenalidomide for 21 days in 35-day cycle for the first cycle, then for 21 days in 28-day cycles for other 6 cycles. During the first cycle rituximab was given 50 mg/m^2^ on day 29, and 325 mg/m^2^ on day 31, 375 mg/m^2^on day 33. During the second cycle rituximab was administered 375 mg/m^2^ weekly and on day 1 during remaining cycles. Early results of the ongoing study suggest that lenalidomide-plus-rituximab immunotherapy is tolerable. The most common grade 3/4 adverse events (AE) were neutropenia (18 pts), anemia (5 pts), and thrombocytopenia (4 pts). There were no cases of neutropenic fever, sepsis, or bleeding. Nonhematologic grade 3/4 AEs included infection (3 pts), rash (2 pts), and pulmonary embolus (2 pts). The protocol was amended to include aspirin prophylaxis. Most frequent AEs (all grades) were the TFR (21 pts), fatigue (19 pts). 

As a consequence of biological and clinical synergism of lenalidomide and anti-CD 20 monoclonal antibody, a combination with the humanized anti-CD20 monoclonal antibody ofatumumab was evaluated in a phase II trial [24]. Ofatumumab was given weekly, starting at 300 mg in the first week, then at 1000 mg weekly, then monthly for 6 months and lastly every 2 months up to 24 months. Lenalidomide was given at a dose of 10 mg daily continuously from day 9 of cycle 1 with treatment duration of 24 months. The ORR was 63% with 2 patients achieving a CR. Toxicity was tolerable, with 50% of patients developing grade 3 or 4 neutropenia. TFR was limited to grade 1 in 2 patients.

In the GIMEMA LLC606 phase I clinical trial patients were treated with lenalidomide, cyclophosphamide, and fludarabine [25]. The maximal tolerated dose of lenalidomide was 5 mg. The response rate observed in nine patients was 67%, with 33% CR.

 Brown et al. [26] initiated a small phase I study that investigated the combination of lenalidomide with fludarabine and rituximab for untreated patients with CLL. A low dose of lenalidomide (2,5 mg) was given daily for 21 days in 28 days cycles, with fludarabine 25 mg/m^2^ on days 3–5 and rituximab 375 mg/m^2^ on day 1. The trial had to be closed due to significant myelotoxicity and idiosyncratic tumor flare reactions. 

Another trial by Egle et al. [27] combined 6 cycles of fludarabine, lenalidomide, and rituximab. Lenalidomide was administered at a starting dose of 2,5 mg daily (days 7–21 in cycle 1) and escalated up to 25 mg/day from days 1–21 of the following cycles. After induction treatment, maintenance with lenalidomide and rituximab for 6 months was planned. Preliminary data show that all ten treated patients achieved at least a PR, except for one patient with Richter transformation; but 50% of patients received a reduced lenalidomide dose due to toxicity.

A phase I study from the German CLL study group [28] used the bendamustine-rituximab (BR) backbone and added lenalidomide to it. This might be an option for patients with relapse or even refractory CLL. Lenalidomide was given orally for 7 days followed by rituximab 375 mg/m^2^ on day 1 in addition tobendamustine90 mg/m^2^ intravenously on days 1 and 2 and lenalidomide orally daily every 28 days for a total of 6 cycles. After 6 cycles of bendamustine, lenalidomide, and rituximab, lenalidomide monotherapy was administered as continued therapy for an additional 6 cycles as tolerated or until disease progression.

Blum et al. [29] conducted a phase I study in which flavopiridol was given in combination with lenalidomide in 21 patients with relapsed or refractory B-cell CLL/SLL. Flavopiridol was administered at 60 mg/m^2^ on days 1, 8, and 15 during first cycle and from second cycle at 60 mg/m^2^ on days 3, 10, and 17 plus, starting at cycle 2, lenalidomide with an initial dose of 2,5 mg escalated to 25 mg for 21 days of these 28-day cycles. Thirteen patients had del(17p) and 8 patients had del (11q). Seventeen patients completed two or more cycles of therapy (median 3, range 2–8), receiving 2,5 mg (6 pts), 5,0 mg (7 pts), and 7,5 mg (4 pts) of lenalidomide. Grade 3-4 toxicities consisted of neutropenia (86%), diarrhea (62%), reversible transaminitis in ≤7 days (57%), anemia (38%), thrombocytopenia (38%), hyperglycemia (38%), infection without neutropenia (24 %), febrile neutropenia (14%), TLS not requiring dialysis (14%), and fatigue (14%). Grade 1 tumor flare occurred in 1 pt and did not require additional steroids or cessation of lenalidomide. The ORR was 46% with no CRs. Partial responses were observed in 7 patients, including 4 patients with del (17p). In conclusion, combined flavopiridol and lenalidomide was well tolerated, with activity in patients with previously treated, cytogenetically high-risk CLL. 

A prospective, nonrandomized, phase II study by Shanafelt et al. [30] presented data about lenalidomide consolidation following a PCR (pentostatin, cyclophosphamide, rituximab) induction. Data from this trial were compared with results of an historic trial of PCR without lenalidomide showing an improvement in quality of response and time to retreatment.

Patients with previously untreated CLL (*n* = 44) received induction therapy with the frontline regimen most commonly used at the Mayo Clinic, pentostatin 2 mg/m^2^, cyclophosphamide 600 mg/m^2^, and rituximab 375 mg/m^2^ every 3 weeks for 6 cycles. Responding patients were eligible for consolidation therapy with lenalidomide at a starting dose of 5 mg/day with escalation to 10 mg/day as tolerated for 6 months. Thirty-four patients (77%) started lenalidomide and completed a median of 7 cycles of consolidation therapy.

 Among patients who started lenalidomide consolidation following PCR, the freedom from retreatment at 12 months was 95% (95% confidence interval: 88% to 100%). The study investigators compared these findings to historic data from a trial of PCR induction therapy without lenalidomide consolidation [31]. In that study of 64 patients with CLL, the freedom from retreatment at 12 months was 86%. Cytopenias were the most frequent grade ≥3 adverse events considered possibly associated with lenalidomide consolidation: grade 3 neutropenia and thrombocytopenia occurred in 41% and 9% of patients, respectively; while grade 4 neutropenia interested 21% of patients. Therefore, the study authors concluded that lenalidomide consolidation appeared to improve the quality of response to induction therapy with PCR.

Another ongoing study which evaluates lenalidomide as maintenance therapy in CLL patients was the CONTINUUM study [32]: a phase 3, multicenter, randomized, double-blind, placebo-controlled, parallel-group study of the efficacy and safety of lenalidomide as maintenance therapy for patients with B-cell chronic lymphocytic leukemia following second-line therapy. In the experimental arm lenalidomide is given on days 1–28 of a 28 day cycle until disease progression or unacceptable toxicity.  Table 2 summarizes the results from clinical trials using lenalidomide in combination regimens in CLL patients. 

## 4. Conclusions

Lenalidomide proved to be effective in CLL as single agents or in combination with various chemo immunotherapeutic regimens. There were several concerns regarding toxicity, but modified protocols with low starting dose and gradual dose escalation suggest good tolerability. Myelosuppression was the predominant toxicity associated with lenalidomide. Tumor flare was also a problem with lenalidomide therapy, but it can be controlled by gradual dose-escalation and prophylactic corticosteroids in the patients who experienced tumor flare in previous cycles. However, further studies are needed to establish the most effective dose and schedule of this agent.

Lenalidomide might have a place in the first-line setting in older patients or as second-third line agent for patients treated with frontline chemoimmunotherapy. Currently, chemoimmunotherapy represents the standard first-line therapy for young and fit CLL patients, but patients who became refractory to fludarabine or carry deletion/mutation of TP53 and older or unfit patients could profit from alternative treatments, including, lenalidomide-based regimens. Therapeutic strategies including consolidation treatment are becoming more important in the treatment of CLL, particularly in the frontline setting. There is a greater number of complete remissions (CRs) with the new chemoimmunotherapy combinations, and therefore prolonging the duration of PFS becomes a more interesting goal. In this context, ongoing promising studies [29, 30] are evaluating the role of lenalidomide as consolidation/maintenance therapy.

## Figures and Tables

**Table 1:** Selected clinical trials using lenalidomide alone for treatment of CLL. NR: not reported.

Study	Regimen	No. of patients	TLS all grades	TFR all grades	Hematologic side effects grade 3/4	OR (%)	CR (%)	OS (%)	PFS (%)
Chanan-Khanet al. [14] Phase II relapsed/refractory CLL	5 mg/d escalated to 25 mg/d	45	5%	58%	Neutropenia 70% thrombocytopenia 45% anemia 18%	47	9	NR	NR

Ferrajoli et al. [6] Phase II relapsed/refractory CLL	10 mg/d escalated to 25 mg/d	44	0	12%	Neutropenia 41% thrombocytopenia 15% anemia 3%	32	7	73 (with a median follow-up time of 14 months)	NR

Chen et al. [16] Phase II untreated CLL	2.5 mg/descalated to 10 mg/d	25	0	88%	Neutropenia 72% thrombocytopenia 28% anemia 20%	56	0	92 (estimated 2 years OS)	89 (estimated 2 years PFS)

Aue et al. [17] Phase II relapsed/refractory CLL	20 mg/d lowered to 10 mg/d	33	0	53%	Neutropenia 56% thrombocytopenia 30% anemia 15%	NR	NR	NR	NR

Badoux et al. [18] phase II (elderly) untreated CLL	5 mg/d escalated to 25 mg/d	60	0	52%	Neutropenia 34% thrombocytopenia 12% anemia <1%	65	10	88 (estimated 2 years OS)	60 (estimated 2 years PFS)

**Table 2:** Selected clinical trials using lenalidomide in combination for treatment CLL. NR: not reported.

Study	Regimen	No. of patients	TLS all grades	TFR all grades	Hematologic side effects grade 3/4	OR (%)	CR (%)	OS (%)	PFS
Ferrajoli et al. [22] phase II relapsed/refractory CLL	10 mg/d Lenalidomide + rituximab weekly	59	1,7%	37%	Neutropenia 68% thrombocytopenia 22% anemia 10%	64	8	NR	NR

Badoux et al. [24] phase II relapsed/refractory CLL	10 mg/d lenalidomide + ofatumumab weekly	16	NR	13%	Neutropenia 50% anemia 13%	63	13	NR	NR

Blum et al. [29] phase I relapsed/refractory CLL	2.5 mg/d escalated to 25 mg/d lenalidomide + flavopiridol	15	14%	7%	Neutropenia 86% thrombocytopenia 38% anemia 38%	46	0	NR	NR

GIMEMA LLC 606 [25] phase I relapsed/refractory CLL	2.5 mg/d escalated to 15 mg/d lenalidomide + cyclophosphamide + fludarabine	9	0	11%	Transient grade 3-4 neutropenia in the majority of pts	67	33	NR	NR

Egle et al. [27] phase untreated CLL	2.5 mg/d escalated to 25 mg/d lenalidomide + fludarabine + rituximab	10	0	0	Neutropenia 70%	90	0	NR	NR
